# Masculinization of Gene Expression Is Associated with Exaggeration of Male Sexual Dimorphism

**DOI:** 10.1371/journal.pgen.1003697

**Published:** 2013-08-15

**Authors:** Marie A. Pointer, Peter W. Harrison, Alison E. Wright, Judith E. Mank

**Affiliations:** 1 Edward Grey Institute, Department of Zoology, University of Oxford, Oxford, United Kingdom; 2 Department of Genetics, Evolution and Environment, University College London, London, United Kingdom; University of Lausanne, Switzerland

## Abstract

Gene expression differences between the sexes account for the majority of sexually dimorphic phenotypes, and the study of sex-biased gene expression is important for understanding the genetic basis of complex sexual dimorphisms. However, it has been difficult to test the nature of this relationship due to the fact that sexual dimorphism has traditionally been conceptualized as a dichotomy between males and females, rather than an axis with individuals distributed at intermediate points. The wild turkey (*Meleagris gallopavo*) exhibits just this sort of continuum, with dominant and subordinate males forming a gradient in male secondary sexual characteristics. This makes it possible for the first time to test the correlation between sex-biased gene expression and sexually dimorphic phenotypes, a relationship crucial to molecular studies of sexual selection and sexual conflict. Here, we show that subordinate male transcriptomes show striking multiple concordances with their relative phenotypic sexual dimorphism. Subordinate males were clearly male rather than intersex, and when compared to dominant males, their transcriptomes were simultaneously demasculinized for male-biased genes and feminized for female-biased genes across the majority of the transcriptome. These results provide the first evidence linking sexually dimorphic transcription and sexually dimorphic phenotypes. More importantly, they indicate that evolutionary changes in sexual dimorphism can be achieved by varying the magnitude of sex-bias in expression across a large proportion of the coding content of a genome.

## Author Summary

Males and females exhibit many differences in morphology, behavior and physiology, yet they share the vast majority of their genomes. Most differences between the sexes are therefore thought to be the product of gene expression differences between females and males. Studies of sex differences in expression assume that genes expressed more in males encode male traits, and genes expressed more in females encode female traits, and this assumption is a key foundation to genetic studies of sexual dimorphism and sexual conflict. Despite this key assumption, this relationship has yet to be empirically tested, as the main model organisms for studies of sex-biased gene expression lack multiple male and female morphs. Here, we use the two male morphs in the wild turkey to show that the magnitude of male-biased gene expression correlates with the manifestation of sexually dimorphic traits. Males with less manifestation of sexual dimorphism in phenotype were both demasculinized for male-biased genes, as well as feminized for female-biased genes. This pattern encompassed the majority of expressed loci, suggesting that evolutionary changes in the magnitude of sexual dimorphism may be achieved by small changes in the magnitude of sex-biased transcription across thousands of genes.

## Introduction

Complex sexually dimorphic phenotypes are largely the result of gene expression differences between males and females for loci that are present in both sexes [1], [2], and the study of sex-biased gene expression provides a link between sexual conflict and sexual selection acting on the phenotype with the genetic loci that underpin it. It is often assumed that genes expressed more in either sex encode sexually dimorphic phenotypes that are then subject to sex-specific selection. Studies in a range of animals have demonstrated that sex-biased gene expression is widespread across the genome [3]–[7], most evident in adults as would be expected as this is when sexual phenotypes are most manifest [8]–[10], variable among closely related species [11] and subject to rates of evolution consistent with sexual selection acting primarily on males [2]. However, despite this mounting circumstantial evidence, the relationship between gene expression and the phenotype is complex, and direct connections linking sex-biased gene expression to sexually dimorphic phenotypes have remained elusive. This relationship between sex-biased transcription and sexual dimorphism is key to studies of sexual conflict and sexual selection, which are increasingly focused on sex-specific regulation, and to the broader question of the regulatory control of complex phenotypes.

The relationship between sex-biased gene expression and sexual dimorphism has been difficult to test directly, primarily because sexual dimorphism is often envisaged as a dichotomous comparison between female and male forms. Additionally, many of the model systems for sex-biased gene expression studies lack multiple sex-specific morphs, precluding detailed tests of the association between sex-biased gene expression and dimorphic phenotypes. However, sexual dimorphism is far more complex for many species, with some individuals occupying intermediate points along an axis. The wild turkey exhibits two male phenotypes in the forms of dominant and subordinate males. The species is strongly sexually dimorphic, with dominant males showing greater body size than females, along with a constellation of sexually selected traits including iridescent plumage, a long beard, vivid coloration on the head and neck, enlargement of the caruncles, wattle and snood (Supplemental Fig. 1), and distinct mating behaviours [12]–[14].

Dominance among sibling males is established via male-male competition during the winter prior to sexual maturation [15], and at this point, many males develop the subordinate male phenotype, which includes iridescent plumage and long beards similar to dominant males, but with less vivid head and neck coloration and less developed wattles, caruncles and snoods. The length of the latter appears to be key to intra-sexual and inter-sexual selection in this species [12], [13]. Although subordinate males can mate and sire offspring [15]–[17] they rarely obtain mating opportunities. Their role is mainly to assist their dominant brothers in attracting mates, and as such has been held up as an example of Hamilton's rule of kin selection [15], [16], [18]. Importantly, subordinate males can become dominant males later in life if the dominant dies, emphasising the plastic nature of the male phenotype. Subordinate males are therefore clearly male in phenotype, but occupy an intermediate position on the continuum of sexual dimorphism.

The two male phenotypes in the wild turkey make it possible to test for the first time whether the magnitude of sexual dimorphism in the phenotype is associated with the magnitude of sex-biased expression. Male-biased genes are often assumed to encode male-specific phenotypes, while female-biased genes are thought to encode female-specific phenotypes. Within this framework, the subordinate male phenotype could be the product of reduced expression of male-biased genes (demasculinized), increased expression of female-biased genes (feminized), or a combination of both, compared to the dominant male phenotype. We therefore used the female and subordinate male and dominant male phenotypes in order to directly test for the first time whether the degree of sex-biased gene expression is correlated with sexual dimorphism, and to understand the role of demasculinization and feminization in gene expression in encoding the subordinate male form.

## Results and Discussion

Our initial preliminary analysis of sex-biased expression indicated, as have previous studies [7], [19], that the gonad is the most transcriptionally dimorphic tissue (Supplemental Table 1), and therefore we focused our analysis primarily on this organ, although we also assessed the spleen using lower fold-change thresholds in order to determine whether the general patterns extend from the gonad to the soma. In the gonad, 9,872 autosomal and 364 Z-linked genes were significantly expressed. Of the autosomal genes, 2,217 were significantly male-biased (dominant male: female fold change >2, adj. *p*<0.05), 2,908 were significantly female-biased (female: dominant male fold change >2, adj. *p*<0.05), and 4,747 were unbiased. The autosomal genes show a broadly similar pattern of sequence evolution to that seen in other adult animals [2], with male-biased genes showing elevated rates of functional evolution compared to female-biased and unbiased genes (Table 1), consistent with the notion that sex-specific selection is stronger in males than females in this species.

**Table 1:** Rates of non-synonymous (d_N_) and synonymous (d_S_) substitution for autosomal sex-biased and unbiased genes.

	Male-biased (n = 1,176)	Female-biased (n = 1,497)	Unbiased (n = 1,156)
d_N_ (95% CI)	0.00685 (0.00578–0.00811)	0.00514 (0.00457–0.00578)	0.00500 (0.00436–0.00574)
d_S_ (95% CI)	0.0506 (0.0450–0.0572)	0.0541 (0.0492–0.0591)	0.0515 (0.0464–0.0569)
d_N_/d_S_ (95% CI)	0.1354 (0.128–0.142)	0.0951 (0.0929–0.0977)	0.0972 (0.0938–0.101)

**Figure 1:**
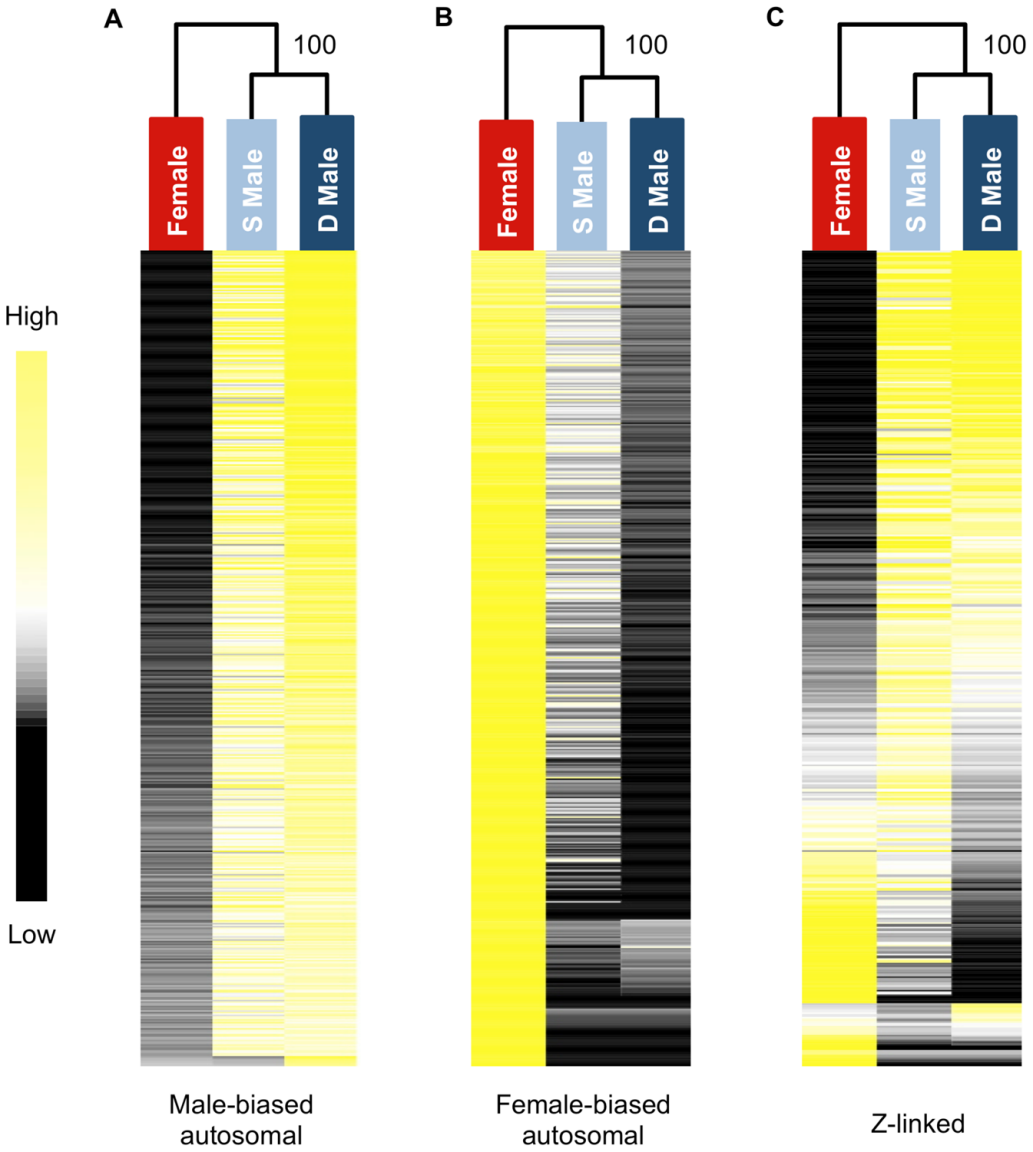
Heat maps and hierarchical clustering of gene expression for females, subordinate males and dominant males. Shown is the relative expression for autosomal male-biased (n = 2,217, panel A), female-biased (n = 2,908, panel B) and Z-linked (n = 364, panel C) genes. Hierarchical gene clustering is based on Euclidean distance for average log_2_ expression for each gene for the three sexual morphs. The number at each node is the percentage bootstrap result from 1000 replicates.

We used hierarchical clustering of expression level to visualize global transcriptomic patterns for the three morphs in the gonad. Subordinate and dominant males clustered together with high confidence for male-biased autosomal, female-biased autosomal and Z-linked genes (Fig. 1). Clustering clearly demonstrates that subordinate male transcription is on the male side of the sexual dimorphism continuum rather than intersex, however there were clear but subtle differences between the male forms in overall transcription that distinguish them. Hierarchical clustering of unbiased autosomal expression also showed the male phenotypes cluster together, with 100% bootstrap support.

**Figure 2:**
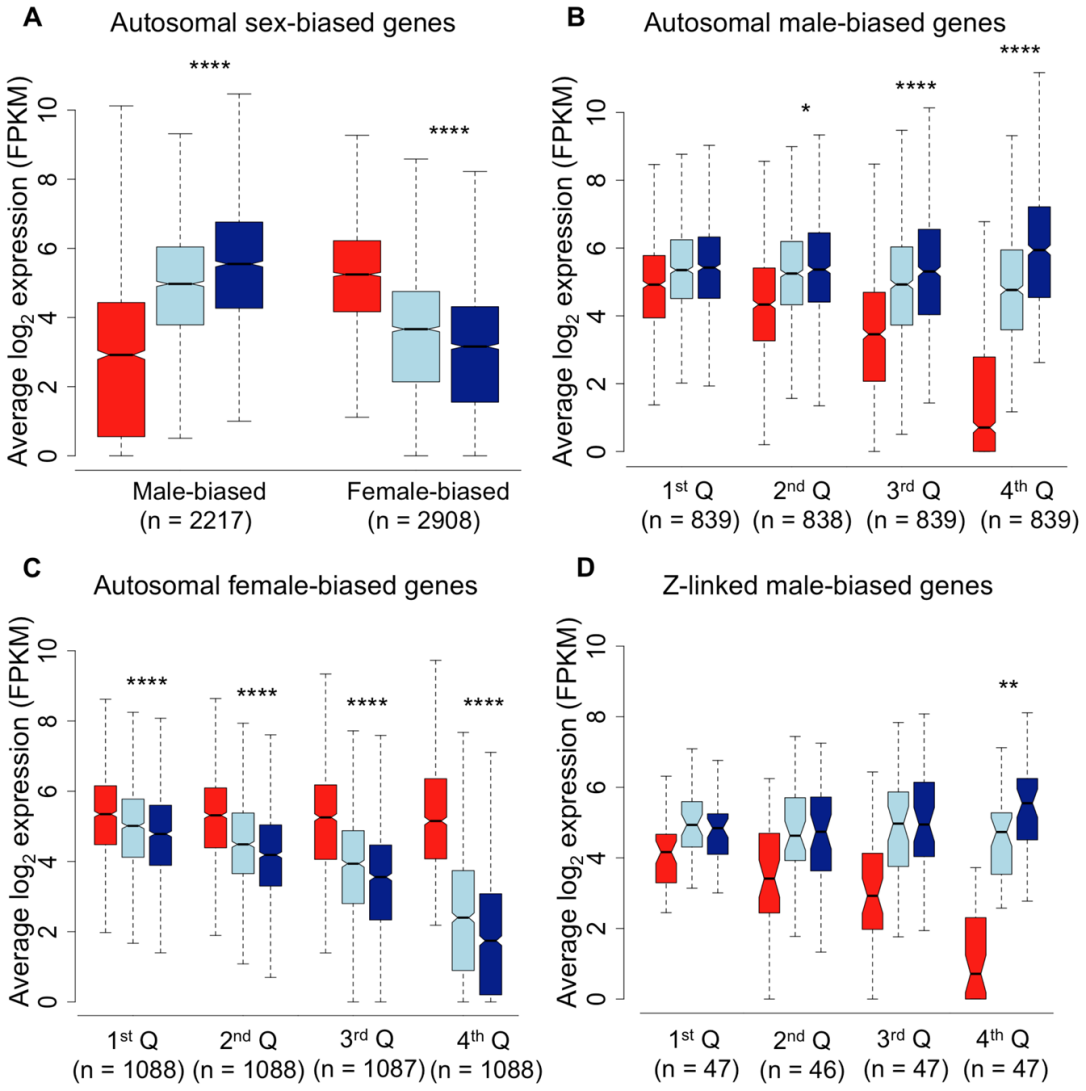
Average log_2_ expression for all sex-biased genes. Panel A, autosomal male-biased and female-biased genes in females (red), subordinate males (light blue) and dominant males (dark blue). Panel B, autosomal male-biased genes ranked by male-bias. Panel C, autosomal female-biased genes ranked by female bias, and Panel D, Z-linked male-biased genes ranked by male-bias. Whiskers extend to the most extreme data point, excluding outliers that exceeded 1.5× the interquartile range. Significant *p*-values as calculated by Wilcoxon tests are indicated by asterisks above each comparison between dominant and subordinate males (* *p*<0.05, ** *p*<0.01, *** *p*<0.001, **** *p*<0.0001).

We next analysed sex-biased genes for evidence of demasculinization and/or feminization in subordinate males in order to examine how sex-biased gene expression is affected by male social dominance (Fig. 2A). Subordinate males express autosomal male-biased genes in the gonad at significantly lower levels than dominant males (Wilcoxon test, *p*<0.00001), suggesting that subordinate males are transcriptionally demasculinized. Just as important is the fact that subordinate males express female-biased genes at a higher level than dominant males (Wilcoxon test, *p*<0.00001, Fig 2A), suggesting that they are transcriptionally feminized.

RNA-Seq data give a relative, rather than absolute measure of expression. It is therefore possible that the relative reduction in expression for male-biased genes in subordinate males could produce a false signal of a relative increase in expression for all other types of genes. In order to ascertain whether the pattern of feminization for female-biased genes was simply an artefact of relative decrease in expression for male-biased genes, we removed all reads mapping to male-biased genes in all three morphs. We remapped the remaining reads, effectively normalizing for differences in expression for male-biased genes. The resulting comparison of unbiased and female-biased genes (Supplemental Fig. 2), suggests that the feminization in transcription in subordinate males is not an artefact of demasculinization.

**Figure 3:**
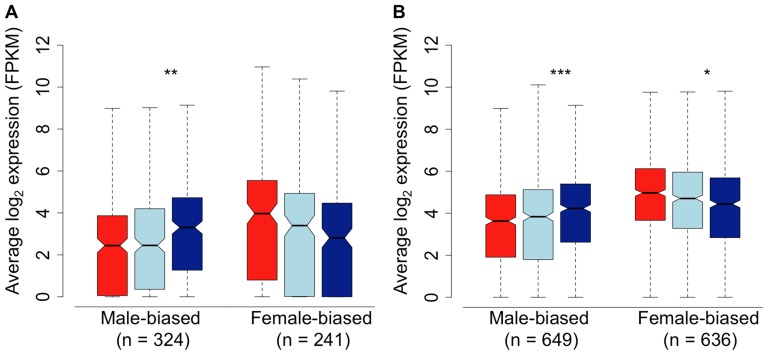
Sex-bias in the spleen of females (red), subordinate males (light blue) and dominant males (dark blue). Sex-bias was defined in panel A based on a 1.5-fold change threshold between females and dominant males, with a p-value<0.05. Sex-bias in panel B is defined solely on statistical difference (*p*<0.05) between females and dominant males. Significant difference between dominant and subordinate males is indicated (Wilcoxon test, * *p*<0.05, ** *p*<0.01, *** *p*<0.001, **** *p*<0.0001).

We assessed gene expression in the spleen, in order to determine whether the pattern we observe in the gonad extends to the soma. Because patterns of sex-bias are much reduced in somatic tissue [4], [7], we relaxed our fold-change thresholds considerably to do this. Despite the lower overall degree of sex-bias, we observed the same qualitative pattern in the spleen compared to the gonad, with subordinate males both demasculinized and feminized in overall transcription compared to dominant males. Despite the fact that the limited overall differences between males and females in transcription in the spleen limits statistical power, the pattern of demasculinization and feminization in the spleen was statistically significant (Wilcoxon test, p<0.05) in three of the four comparisons (Fig. 3). This suggests that the pattern of demasculinization and feminization is not limited to the gonad, but extends into the soma as well, although to a lesser degree.

In order to further test whether subordinate males are intermediate, or orthogonal, to dominant males and females in overall expression, we performed factor analysis for all expressed genes in the gonad and spleen. In both tissues, subordinate males are clearly more similar to dominant males, although intermediate between dominant males and females (Supplemental Fig. 3). This is congruent with the concept that the three sexual morphs form an axis of dimorphism.

The overall pattern of demasculinization and feminization of subordinate male transcription is strongly correlated with the degree of sex-bias. Demasculinization of male-biased gene expression in subordinates is more pronounced for genes with greater male-bias in the gonad (significance for each quartile is denoted in Fig. 2B), possibly suggesting that the most extreme male-biased genes make the greatest contribution to male-specific traits. Similarly, feminization increases for genes with greater female-bias (Fig 2C), indicating feminization of the subordinate male transcriptome for female-biased genes. We lacked sufficient sex-biased genes in the spleen to do a meaningful quartile-based analysis. We calculated the overall correlation between the difference in transcription between dominant and subordinate males (log_2_ dominant male expression – log_2_ subordinate male expression) with the degree of female-bias. This analysis recovered a significant correlation for both the gonad-expressed genes (r^2^ = 0.307, *p*<0.001) and the spleen (r^2^ = 0.159, *p*<0.001), indicating that as sex-bias increases, subordinate and dominant male transcription is increasingly decoupled in both tissues.

In order to assess whether the patterns we observe are artefacts of the way in which we defined sex-bias, we further examined the gonad data, where sex-bias is most evident. Our results indicate that demasculinization and feminization of subordinate male transcriptomes is independent of how sex-bias is defined. Qualitatively similar patterns are evident when sex-bias is defined by comparing female expression to the combined dominant and subordinate male expression or to subordinate male expression alone (Supplemental Fig. 4). We also tested for the possible influence of regression toward the mean by randomizing samples, in each case picking three dominant male and three female samples to define sex-bias (greater than two-fold expression difference, adj. *p*<0.05), and then assessing the remaining dominant males, females and subordinate males for average expression for female-biased and male biased genes. There was no difference between female sample groups or between dominant male sample groups (Wilcoxon test, all *p*>0.05 after Bonferroni adjustment for multiple comparisons) in any of the 100 sample combinations. In every case, subordinate male expression was significantly different than dominant male expression (Wilcoxon test, *p*<0.05 after Bonferroni adjustment for multiple comparisons). We also randomized our definition of female-bias for the renormalized dataset which corrects for any artefacts of differences in male-biased expression (Supplemental Fig. 2). As with the full dataset there was no difference in female-biased genes between female sample groups or between dominant male sample groups in any of the sample combinations (Wilcoxon test, all *p*>0.05 after Bonferroni adjustment for multiple comparisons), and subordinate male expression was significantly different to dominant male expression in all but one of the combinations (Wilcoxon test, *p*<0.05 in 99 out of 100 comparisons after Bonferroni adjustment for multiple comparisons). Additionally, increasing male-bias was largely due to a reduction in expression in females rather than an increase in male expression. Similarly, increasing female-bias was primarily due to reduced male expression rather than an increase in females (Fig. 2 and Supplemental Fig. 5).

We also randomized the spleen data in order to test whether the intermediate position of subordinate males was due to regression toward the mean. There was no evidence of regression toward the mean for male- or female-biased genes between dominant male sample groups in any of the sample combinations (Wilcoxon test, all *p*>0.05 after Bonferroni adjustment for multiple comparisons), and subordinate male expression was significantly different to dominant male expression in all combinations (Wilcoxon test, all *p*<0.05 after Bonferroni adjustment for multiple comparisons). Due to the limited number of samples it was only possible to iterate on dominant males and not on female samples.

Because the Z chromosome is thought to play an important role in male sexually selected traits [20], and because incomplete dosage compensation on the avian Z chromosome results in an average male-bias of Z chromosome expression [21], we assessed Z-linked loci separately. Z-linked male-biased genes in the gonad gave the same result as seen for male-biased autosomal genes, with the most male-biased quartile showing significantly lower expression in subordinate males (Fig. 2D). However the pattern overall was not exaggerated compared to the autosomes, as might be expected if the Z chromosome represented a hotspot for genes encoding male sexually selected traits. We also regressed the magnitude of difference in expression between dominant and subordinate males against male-biased expression for all autosomal and Z-linked male-biased genes separately. The slope of each regression was 0.32 (95% CI = 0.33-0.30) and 0.35 (95% CI = 0.42-0.29) for autosomal genes and Z-linked genes respectively. The overlapping confidence intervals suggest that there is no significant difference between the two slopes, and that the Z chromosome does not play a larger role than expected in encoding the differences between dominant and subordinate males.

Although this could potentially be due to the reduced gene number of the Z chromosome, it also may suggest that the Z chromosome does not play a disproportionately large role in encoding the male sexually selected traits that differ between dominant and subordinate males, but rather its effect is in proportion to its relative size. We did not assess female-biased Z-linked genes as the lack of dosage compensation in birds means there are very few genes that fit these criteria.

Our results show that as genes become more sex-biased, the difference between subordinate and dominant male average expression increases, with subordinate males expressing the most male-biased genes at a lower level and the most female-biased genes at a higher level than their dominant counterparts. The results from male-biased and female-biased genes are the first evidence that subordinate male turkeys show both demasculinization (expressing male-biased genes less) and feminization (expression female-biased genes more) in overall expression patterns that are remarkably concordant with their phenotypic status. This pattern is most evident in the gonad, where sexual dimorphism in transcription is the greatest. However, we observe a similar pattern, although to a lesser degree, in the spleen, suggesting that the concordance between phenotypic sexual dimorphism and transcriptional dimorphism extends to the soma as well.

**Figure 4:**
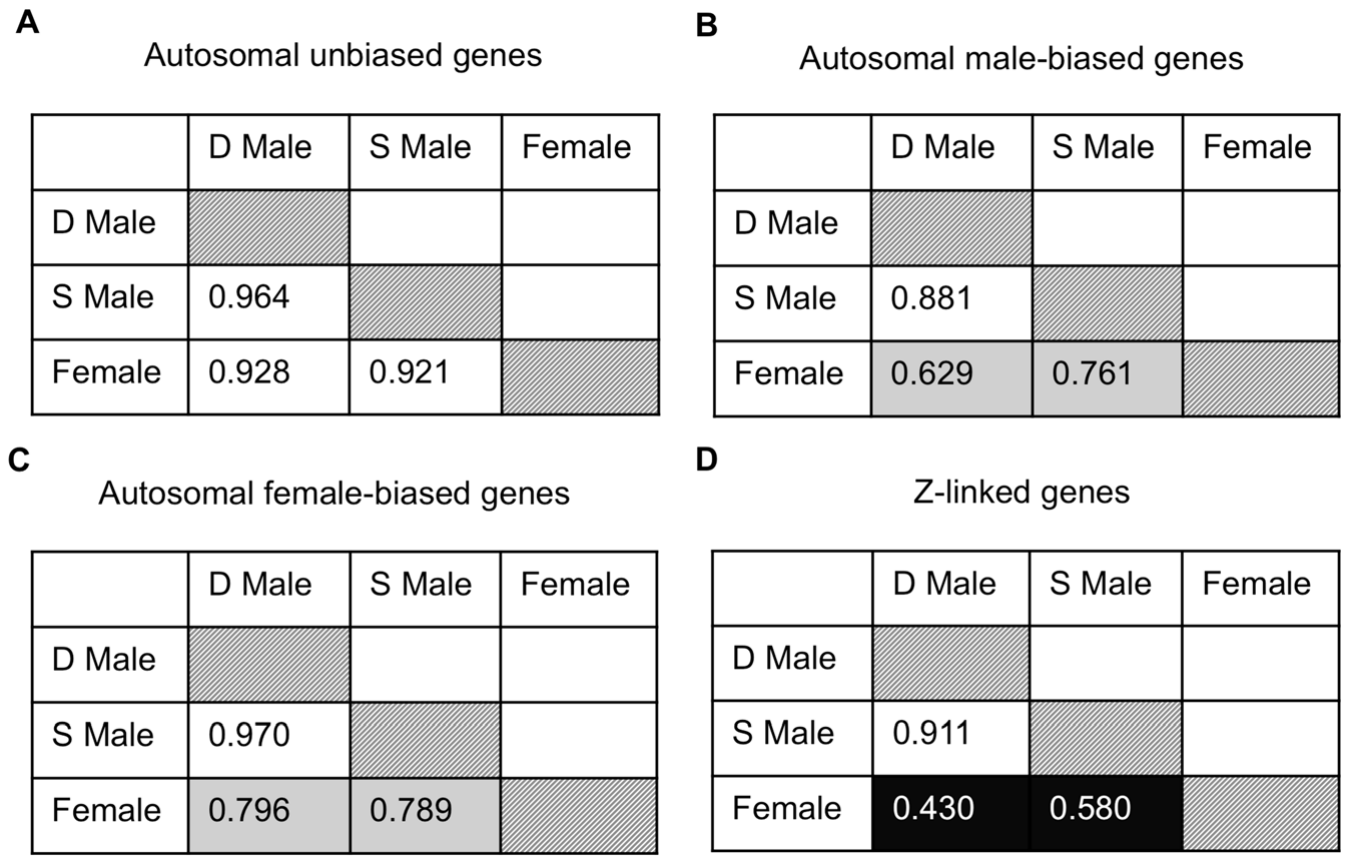
Expression similarity across sexual forms. Spearman rank order (ρ) correlations for average expression for females, subordinate males and dominant males for autosomal unbiased (panel A), autosomal male-biased (panel B), autosomal female-biased (panel C), and Z-linked (panel D) genes. Correlation values are colour coded with lighter colours indicating greater correlation.

Given the pattern of demasculinization, we might expect reduced correlation between dominant and subordinate expression for male-biased genes on the autosomes and Z chromosome compared to other types of genes, as the erosion of intersexual correlation is one way that conflict over optimal transcription can be resolved [22]. We therefore performed Spearman rank correlations on female, dominant and subordinate male average expression for autosomal unbiased, male-biased and female-biased, as well as Z-linked, genes expressed in the gonad (Fig. 4). Male-biased autosomal genes showed a lower correlation between dominant and subordinate males (ρ = 0.881) than for female-biased (ρ = 0.970) or unbiased genes (ρ = 0.964), and this was significant (Fisher r-to-z transformation male-biased v. female-biased *p*<0.0001 and male-biased v. unbiased *p*<0.0001). Also, for Z-linked and male-biased genes, there is greater correlation between subordinate male and female expression (ρ = 0.580 for Z-linked genes, and ρ = 0.761 for autosomal male-biased genes) than is found between dominant male and female expression (ρ = 0.430 for Z-linked and ρ = 0.629 for male-biased autosomal genes, Fisher r-to-z transformation male-biased *p* = 0.000, Z-linked *p* = 0.007, unbiased *p* = 0.018, female-biased *p* = 0.472). Both these results support our prediction that subordinate and dominant males are more divergent for those genes under the greatest male-specific selection, i.e. male-biased autosomal and Z-linked genes, with subordinate male expression showing evidence of demasculinization for male-biased autosomal and Z-linked genes (which are also largely male-biased). Interestingly, the correlation between either male form with females was roughly half for Z-linked genes compared to autosomes, and this may be in part due to incomplete dosage compensation in birds [21].

**Figure 5:**
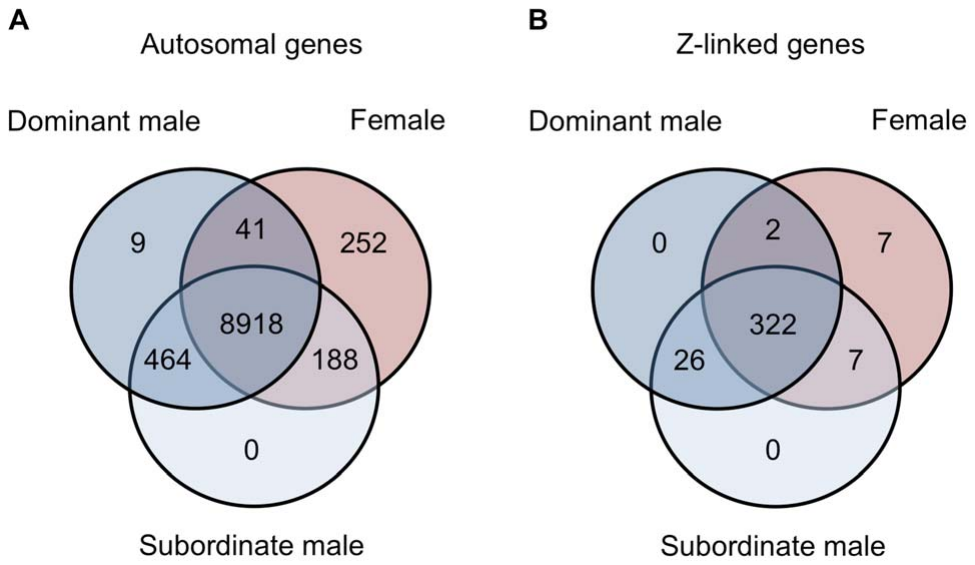
Genes shared between morphs. Venn diagrams for the number of autosomal (panel A) and Z-linked (panel B) genes expressed in females (red), subordinate males (light blue) and dominant males (dark blue).

Finally, we examined gene expression in the gonad within each phenotype. Of the 9,872 autosomal expressed genes, 8,918 (90.3%) were expressed to some degree in all three phenotypes, 252 were female-limited and 473 were male-limited (Fig. 5A). Of the latter, only 9 were limited to dominant males and the remainder were present in both male forms. Interestingly, females and subordinate males shared more than four times as many genes (188) as did females and dominant males (41) (Z-test *p*<0.00001). The same pattern was evident for Z-linked genes, although this was not statistically significant (Fig. 5B, Z-test *p* = 0.098). This suggests that although subordinate males share the greatest expression overlap with dominant males, they show greater similarity to females than do dominant males. There were no GO term enrichments for the genes shared between dominant males and females, and over-abundant GO terms for the genes shared between female and subordinate male turkeys and between dominant and subordinate male turkeys are listed in the supplemental materials (Supplemental Tables 2–3).

Although our results indicate that dominant and subordinate males differ subtly across the transcriptome, they also differ substantially for 21 genes (all autosomal) in the gonad that were significantly differentially expressed between subordinate and dominant males (fold change >2, adj. *p*-value<0.05, Supplemental Table 4), and there was no significant enrichment of Gene Ontology (GO) terms in this gene list. There were no genes that were statistically significant between male morphs in the spleen. The locus with the greatest expression bias toward subordinate males in the gonad, *cytochrome P450, family 11, subfamily A, polypeptide 1* (*CYP11A1*), encodes a catalytic enzyme involved in the first and rate-limiting step in the steroid hormone biosynthesis pathway [23], [24]. Variation in *CYP11A1* has been linked to serum testosterone levels [25], [26], and although this might suggest that testosterone is directly associated with the observed differences in subordinate and dominant male transcription, we found no association between male-bias and proximity to testosterone binding motifs, and the nearby presence of testosterone binding motifs does not explain the expression differences between subordinate and dominant males (Supplemental Tables 5–6). Although it has been suggested that the relative paucity of testosterone binding domains acts as a brake on the evolution of sexual dimorphism [27], our analysis suggests that sex-biased gene expression is controlled by a more complicated regulatory system.

### Conclusions

Our analyses provide the first correlative support linking magnitude of sex-biased gene expression to the degree of phenotypic sexual dimorphism. Our data show a clear and strikingly direct concordance between relative expression of male sexually selected traits and transcriptional masculinization and feminization at multiple levels, in both the gonad and the soma. Synthesis and decay rates can differ for transcription and translation, which can break down the correlation between mRNA abundance and protein titer. However, in some studies, up to 70% of the variance in protein abundance is explained by mRNA levels [28]. Additionally, the broad, genome wide pattern we observe suggests that many of the differences in gene expression levels between male morphs will have functional consequences.

It is not clear whether this axis of dimorphism extends to systems with alternative male mating strategies, as observed in some fish species, where sneaker males and female mimics seek to steal fertilization events from dominant males. In these cases, males with alternative morphs likely divert effort from sexually selected somatic traits to reproductive function and sperm production [29], and so it is difficult to predict what we might expect in transcriptomic comparisons. However, our results suggest that evolutionary changes in the magnitude of sexual dimorphism, which affect a large number of species in many clades, may be achievable by changes in the magnitude of sex-biased transcription.

## Materials and Methods

Two-year-old wild turkeys were obtained in the breeding season of their first reproductive year, after social dominance was established, from Vicvet Farms (Yorkshire, UK). Although the population is natural in that is has not been subject to selection for domestication traits, it is kept under controlled semi-natural conditions, allowing us to control for age, diet and many environmental influences that can potentially affect gene expression. All samples were collected under permission from institutional ethical review committees and in accordance with national guidelines. In each case, the telencephalon, spleen and left gonad were collected separately, homogenized and stored in RNAlater. RNA was prepared from the same volume of starting material with the Animal Tissue RNA Kit (Qiagen). Library and RNA-Sequence samples were prepared and barcoded by the Wellcome Trust Centre for Human Genetics, University of Oxford, using standard methods and sequenced on an Illumina HiSeq 2000 as paired-end 100 bp reads.

The resulting data was assessed for quality using FastQC (http://www.bioinformatics.bbsrc.ac.uk/projects/fastqc). Trimmomatic [30] was used to remove read pairs with residual adaptor sequence and conduct quality filtering. Reads were trimmed if the leading or trailing bases had a Phred score <4, and were also trimmed if a sliding window average Phred score over four bases was <15. Post filtering, reads where either pair was <25 bases in length were removed from subsequent analyses, leaving on average more than 26 million mappable paired-end reads per sample.

The genome of *Meleagris gallopavo* [31] version 2.01 (GCA_000146605.1), was obtained from Ensembl release 67 [32]. Filtered reads were mapped to the genome (excluding rRNA regions) using RSEM, version 1.1.20 [33], which leverages the short-read aligner bowtie, version 0.12.8 [34]. To remove non- and lowly-expressed genes, a minimum expression filter of four reads per million mappable reads was applied to the raw counts, as we have previously implemented for deep RNA-Seq datasets [35]–[36]. All genes expressed lower than this threshold in less than half the female, dominant male or subordinate male individuals were removed from further analysis to prevent our results being biased by the noise inherent in very lowly expressed genes. Fragments per kilobase per million mappable reads (FPKM), which corrects for variations in contig length and read depth between samples was calculated from these raw counts for each sample [37].

To explore the expression differences among the three sexual phenotypes in the gonad, we calculated average log_2_ expression for all females, dominant males and subordinate males for each gene, and tested for sex-bias in several ways using the R package, DESeq [38], which calculates differential expression in a pairwise fashion by negative binomial modelling and adjusts for multiple testing using the Benjamini-Hochberg method. For the gonad, we first tested for sex-bias by identifying significant expression differences (>2-fold difference, p<0.05) between females and dominant males. However, in order to verify that our results were not artefacts of how we defined sex-bias, and regression toward the mean, we also identified those genes with significant expression differences between females and subordinate males, and between females and all males. Due to the reduced level of transcriptional dimorphism in the soma, we reduced our fold-change thresholds considerably for the spleen (Supplemental Materials).

We performed hierarchical clustering using Euclidean distance with complete linkage, as implemented in Cluster 3.0 [39] and visualized in TreeView (v.1.1.6) [40]. Heat maps were separately constructed for male-biased, female-biased and unbiased autosomal genes and Z-linked genes. The reliabilities of the inferred trees were tested by bootstrap resampling (1000 replicates) using the R package, Pvclust [41].

We separated autosomal and Z-linked genes for two reasons. First, the sex chromosomes in birds show incomplete dosage compensation [21], therefore they exhibit an overall male-bias due to gene dose effects. Additionally, the unbalanced sex-specific selection acting on the sex chromosomes has been shown in chicken to masculinize Z chromosome expression [42]–[43]. These patterns mean that although the Z chromosome is interesting in its own right, it cannot be directly compared in terms of sex-bias to the autosomes. Therefore, sex-bias for autosomal genes was defined as those genes expressed two-fold higher in dominant males or females, with an adjusted *p*-value<0.05 (unpaired t-test, Benjamini-Hochberg correction for multiple comparisons [44]). Unbiased genes were all those not classified as either male- or female-biased. When average log_2_ expression values for quartiles based on sex-bias were calculated, the fold change criteria was dropped so as to include genes with a lower fold change than 2. This prevented restriction of the quartile analysis to solely the most sex-biased genes but allowed comparison to genes differentially expressed between the sexes but sex-biased to a lesser degree.

GO term enrichment analysis was performed by taking mouse Ensembl gene IDs for those genes with a 1∶1 mouse ortholog, identified via Biomart. The target list (i.e. 21 significantly differentially expressed genes between dominant and subordinate males, or genes shared between two morphs) were compared to a background list (either all expressed autosomal genes or all expressed genes) using Gorilla [45]–[46]. *P*-values were calculated using a hypergeometric model and corrected for multiple testing.

In order to investigate d_N_ and d_S_, the turkey genome was compared to the genomes of chicken (*Gallus gallus*) and zebra finch (*Taeniopygia guttata*), obtaining 16,496, 22,194 and 18,204 peptides and corresponding cDNA sequence for each species respectively from Ensembl. Proteinortho [47], with default parameters, was used to identify single copy orthologs held in all three species. These 7,854 orthologous groups were aligned with PRANK using a guide tree obtained from Superfamily 1.75 [48]. This orthologous set was filtered with Repeatmasker (http://www.repeatmasker.org) to remove seven retrotransposons and perl scripts were used to remove two genes with in frame stop codons and 13 genes with less than 100 bp in aligned gapless length. PAML, version 4.4b [49], was used to analyse the remaining 6,839 one-to-one orthologs, utilizing the phylogeny used for PRANK above. Alignments where d_S_>2 were removed as this represents the point of mutational saturation in avian sequence data [50]. For those alignments that passed filtering, the number of potential nonsynonymous substitutions (NdN), the number of nonsynonymous substitutions (N), the number of potential synonymous substitutions (SdS) and the number of synonymous substitutions (S) were extracted for each orthologous group for the turkey-specific branch of the three-species phylogeny. These values were summed for each expression category in order to calculate average d_N_ and d_S_ for male-biased, female-biased and unbiased genes. This has the advantage of simultaneously avoiding the problem of infinitely high d_N_/d_S_ values for genes lacking synonymous substitutions while weighting the data by alignment length [9].

The location of androgen transcription factor binding sites (tfbs) in the turkey genome were predicted using amniote androgen tfbs motifs [51]. The predicted tfbs locations were then compared to the start sites of all turkey genes in 2 kb, 5 kb and 10 kb upstream windows and matching hits recorded.

## Supporting Information

Figure S1 Male and female sexual dimorphisms in *Meleagris gallopavo*. Females are smaller than males, and lack both beards and iridescent plumage. In addition to size and plumage differences, males exhibit more vivid coloration on the head and neck, elongated snoods, enlarged caruncles, and a larger wattle or dewlap.
(PDF)

Figure S2 Expression differences between sexual morphs for unbiased and female-biased autosomal genes. Panel A. Average expression for autosomal unbiased genes in females, subordinate males, and dominant males. The increase (2.76%) in average expression between subordinate and dominant male morphs is less than the decrease observed for male-biased (11.35%) or the increase observed for female-biased (15.74%) autosomal genes. Panel B. Relative expression for autosomal unbiased and female-biased genes, correcting for relative differences in male-biased expression between male morphs. FPKM was calculated after removing reads mapping to male-biased genes from the total pool of reads for all samples. This eliminates any potential bias in the remainder of the data due to read differences between male morphs in male-biased genes. Statistical difference between subordinate and dominant male expression is indicated with asterisks (Wilcoxon test, * *p*<0.05, ** *p*<0.01, *** *p*<0.001, **** *p*<0.0001).
(PDF)

Figure S3 Factor analysis of gonad and spleen average gene expression for females, subordinate males and dominant males. Shown are the first two factors accounting for 48.2% and 33.7% of the variance respectively. Factor analysis was performed using the R package ‘factanal’. Suitability of the data for factor analysis was confirmed with a Kaiser-Meyer-Olkin factor >0.83, a significant Bartlett's test of sphericity (chi-square 417293.7, *p*<0.00001) and the factorability of the dataset with correlation of all samples above 0.5. Three factors were selected for the analysis using a 95% cumulative variance cut-off.
(PDF)

Figure S4 Expression differences between sexual morphs is not dependent upon how sex-bias is defined. Panel A. Expression differences between sexual morphs for autosomal sex-biased genes where sex-bias is defined as those genes expressed two-fold higher in subordinate males or females, with an adjusted *p*-value<0.05. Statistical difference between subordinate and dominant male expression is indicated with asterisks (Wilcoxon test, * *p*<0.05, ** *p*<0.01, *** *p*<0.001, **** *p*<0.0001). Panel B. Expression differences between sexual morphs for autosomal sex-biased genes where sex-bias is defined as those genes expressed two-fold higher in all males or females, with an adjusted *p*-value<0.05. Statistical difference between subordinate and dominant male expression is indicated with asterisks (Wilcoxon test, * *p*<0.05, ** *p*<0.01, *** *p*<0.001, **** *p*<0.0001).
(PDF)

Figure S5 Expression level and sex-bias. Relationship between expression level and sex-bias for male-biased genes in dominant males (panel A), subordinate males (panel B) and females (panel C). Relationship between expression level and sex-bias for female-biased genes in dominant males (panel D), subordinate males (panel E) and females (panel F). Pairwise tests of significant difference between quartiles are denoted with letters, shared letters indicate that quartiles within a panel are not significantly different (Wilcoxon test, *p*<0.05). Spearman rank order correlations are given for each panel.
(PDF)

Table S1 Number of sex-biased autosomal genes expressed in the spleen, brain and gonad of the turkey. Genes are sex biased if they are expressed at least two-fold higher in one sex with an adj. *p*-value<0.05.
(DOCX)

Table S2 List of significantly different GO terms for genes shared between female and subordinate male turkeys. GO term enrichment analysis for 195 genes shared between female and subordinate males.
(DOCX)

Table S3 List of significantly different GO terms for genes shared between dominant and subdominant male turkeys. GO term enrichment analysis for 490 genes shared between dominant and subordinate males.
(DOCX)

Table S4 List of genes differentially expressed between subordinate and dominant male turkeys. Binomial *p*-values, adjusted for multiple comparisons, were calculated in DESeq. An asterisk indicates the single differentially expressed gene found to lie within 10 kb of a testosterone receptor binding site.
(DOCX)

Table S5 Number and percentage of genes that reside in close proximity to at least one testosterone DNA binding motif and are male-biased. *P*-values are calculated using a two-sided Z-test by comparison to the actual number of male-biased genes in the genome (2217, 22.46%).
(DOCX)

Table S6 Average log_2_ fold change between subordinate and dominant males for genes that reside in close proximity to at least one predicted testosterone DNA binding motif. Significant difference from overall average log_2_ fold change (0.00044) was calculated by a permutation test with 1000 replicates.
(DOCX)
