# Supplementary figures and images for: Masculinization of Gene Expression Is Associated with Exaggeration of Male Sexual Dimorphism
Source: PLoS Genet. 2013 Aug 15;9(8):e1003697. doi: 10.1371/journal.pgen.1003697 (PMC3744414; doi:10.1371/journal.pgen.1003697)

Female

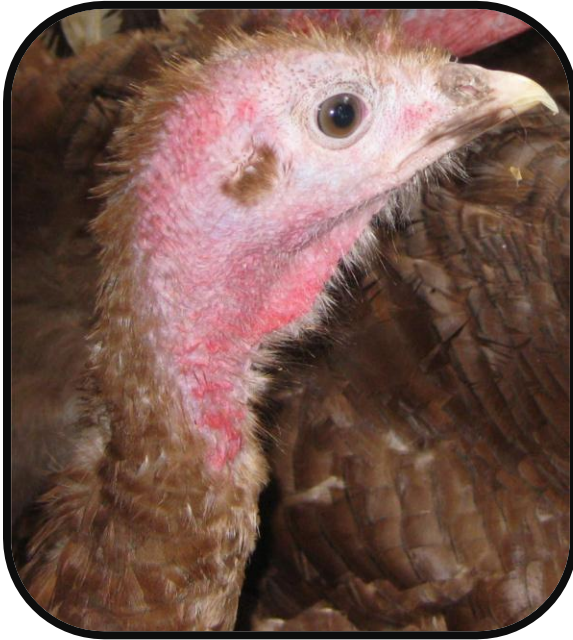

Male

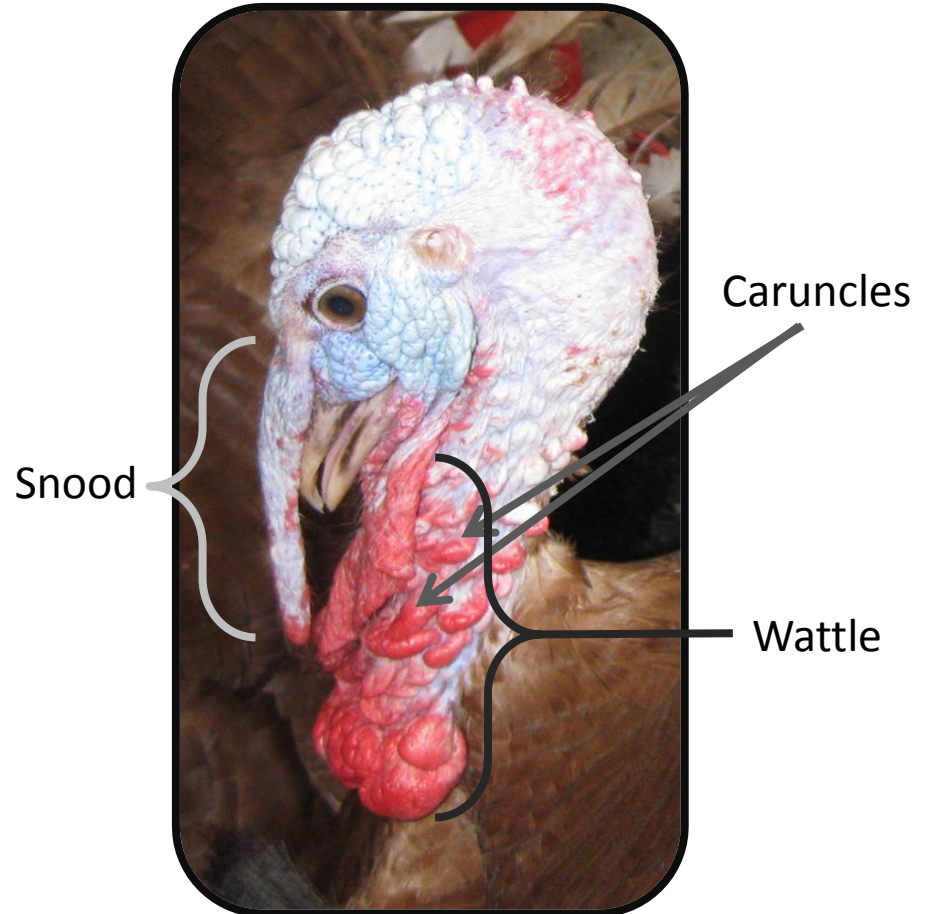

Supplement: Figure S1 — Male and female sexual dimorphisms in Meleagris gallopavo. Females are smaller than males, and lack both beards and iridescent plumage. In addition to size and plumage differences, males exhibit more vivid coloration on the head and neck, elongated snoods, enlarged caruncles, and a larger wattle or dewlap. (PDF) [file pgen.1003697.s001.pdf]

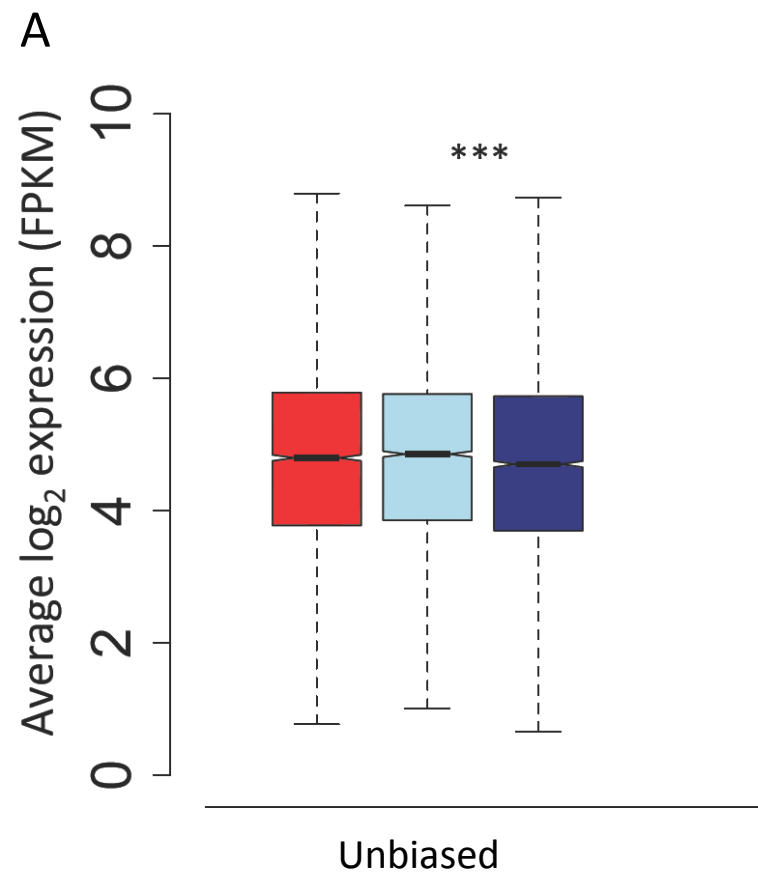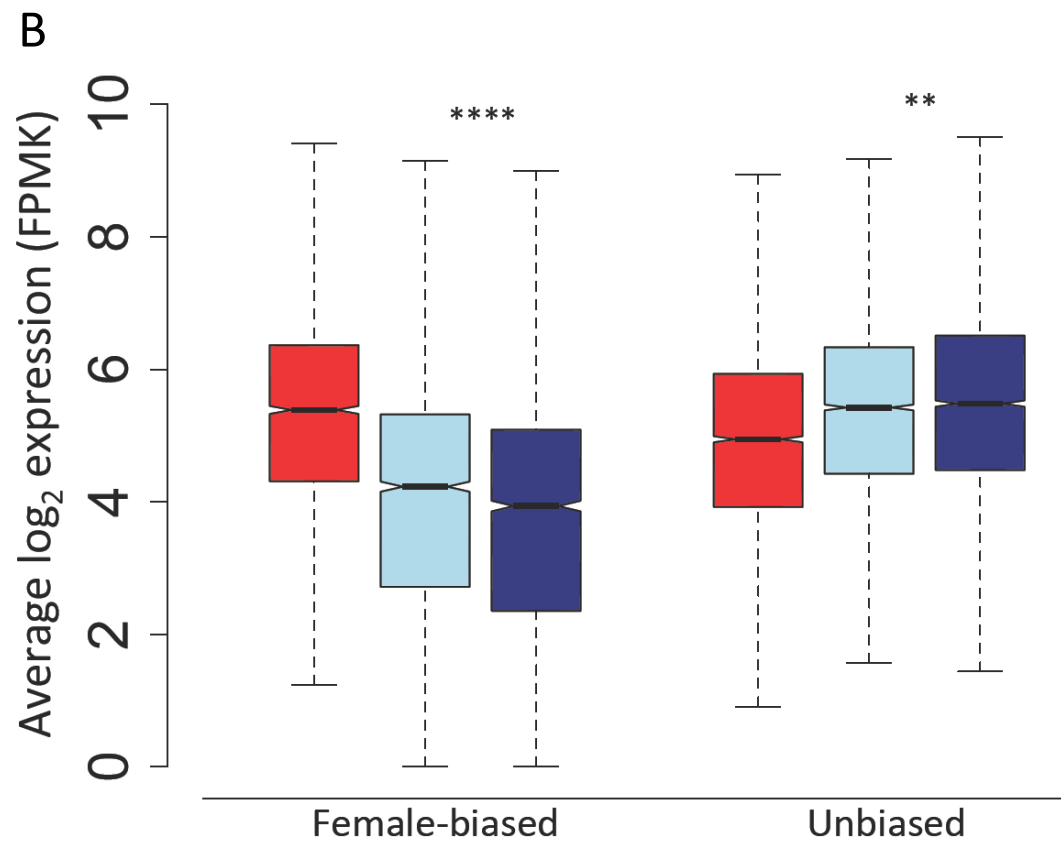

Supplement: Figure S2 — Expression differences between sexual morphs for unbiased and female-biased autosomal genes. Panel A. Average expression for autosomal unbiased genes in females, subordinate males, and dominant males. The increase (2.76%) in average expression between subordinate and dominant male morphs is less than the decrease observed for male-biased (11.35%) or the increase observed for female-biased (15.74%) autosomal genes. Panel B. Relative expression for autosomal unbiased and female-biased genes, correcting for relative differences in male-biased expression between male morphs. FPKM was calculated after removing reads mapping to male-biased genes from the total pool of reads for all samples. This eliminates any potential bias in the remainder of the data due to read differences between male morphs in male-biased genes. Statistical difference between subordinate and dominant male expression is indicated with asterisks (Wilcoxon test, * p<0.05, ** p<0.01, *** p<0.001, **** p<0.0001). (PDF) [file pgen.1003697.s002.pdf]

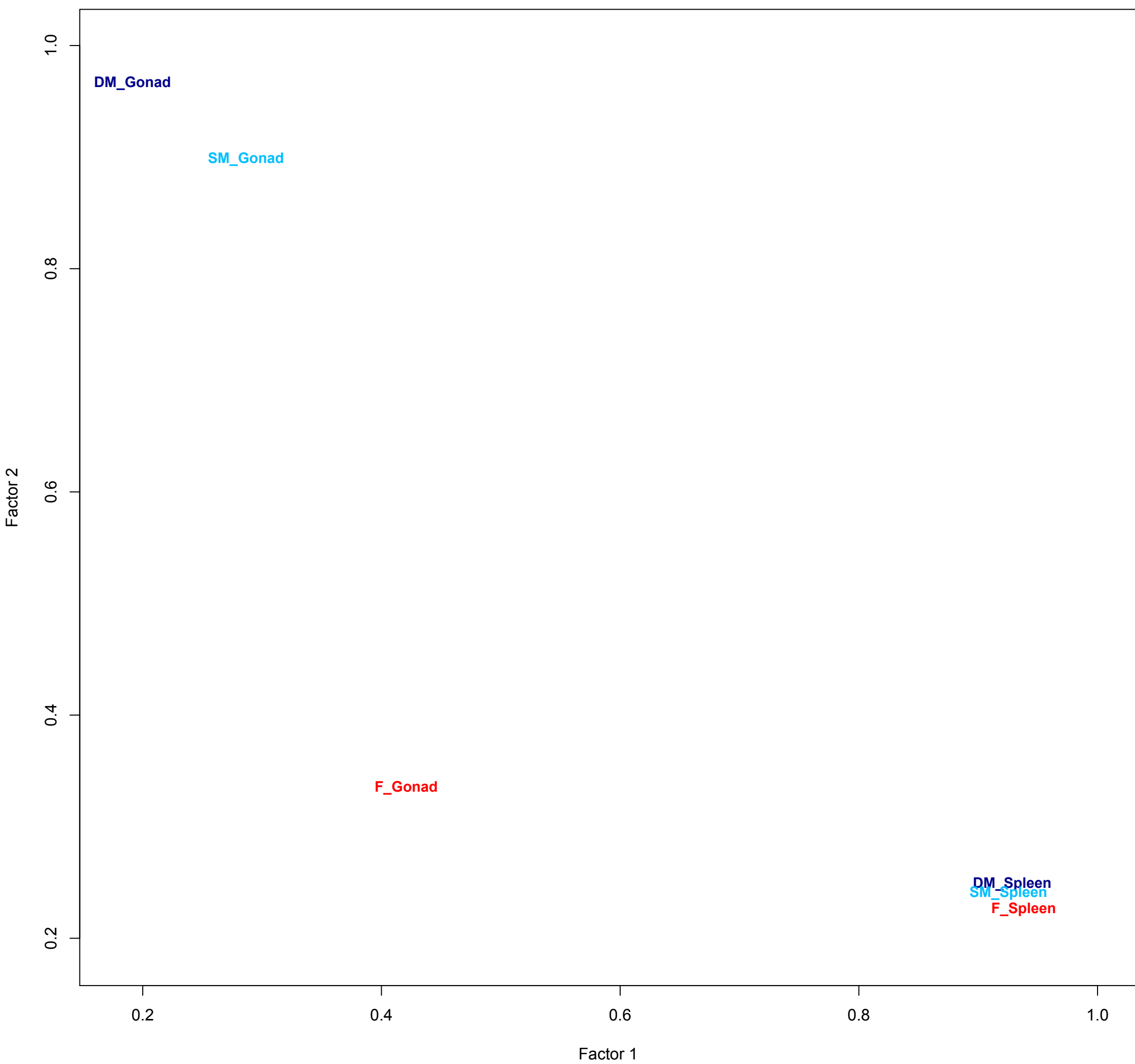

Supplement: Figure S3 — Factor analysis of gonad and spleen average gene expression for females, subordinate males and dominant males. Shown are the first two factors accounting for 48.2% and 33.7% of the variance respectively. Factor analysis was performed using the R package ‘factanal’. Suitability of the data for factor analysis was confirmed with a Kaiser-Meyer-Olkin factor >0.83, a significant Bartlett's test of sphericity (chi-square 417293.7, p<0.00001) and the factorability of the dataset with correlation of all samples above 0.5. Three factors were selected for the analysis using a 95% cumulative variance cut-off. (PDF) [file pgen.1003697.s003.pdf]

A

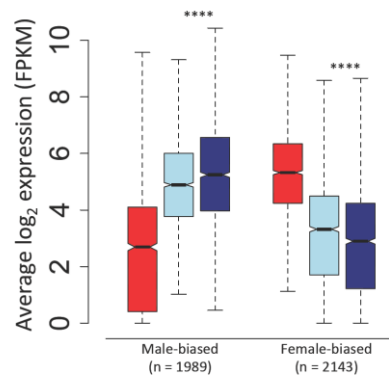

B

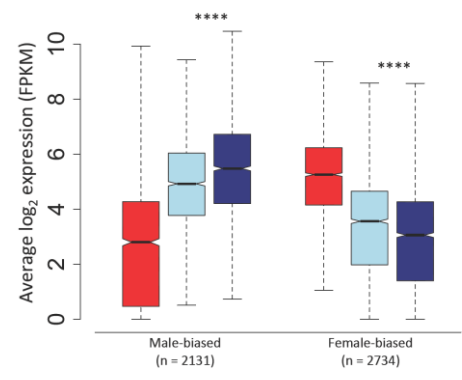

Supplement: Figure S4 — Expression differences between sexual morphs is not dependent upon how sex-bias is defined. Panel A. Expression differences between sexual morphs for autosomal sex-biased genes where sex-bias is defined as those genes expressed two-fold higher in subordinate males or females, with an adjusted p-value<0.05. Statistical difference between subordinate and dominant male expression is indicated with asterisks (Wilcoxon test, * p<0.05, ** p<0.01, *** p<0.001, **** p<0.0001). Panel B. Expression differences between sexual morphs for autosomal sex-biased genes where sex-bias is defined as those genes expressed two-fold higher in all males or females, with an adjusted p-value<0.05. Statistical difference between subordinate and dominant male expression is indicated with asterisks (Wilcoxon test, * p<0.05, ** p<0.01, *** p<0.001, **** p<0.0001). (PDF) [file pgen.1003697.s004.pdf]

**A**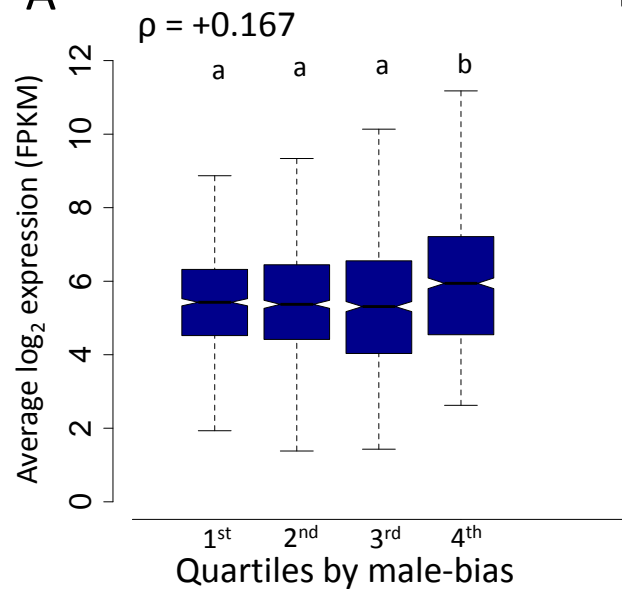**B**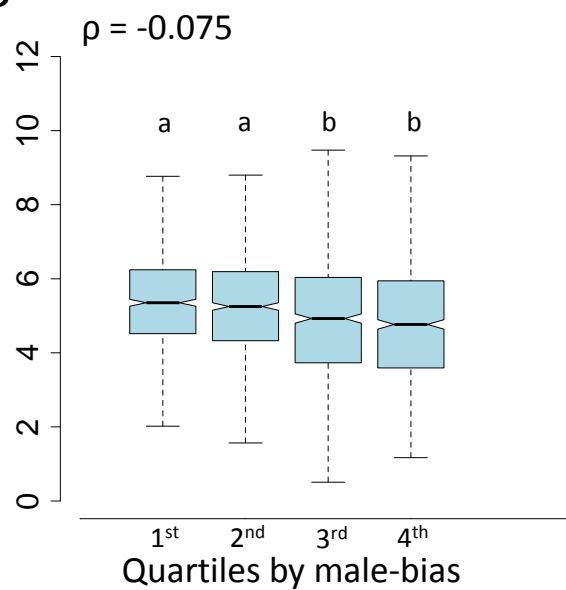**C**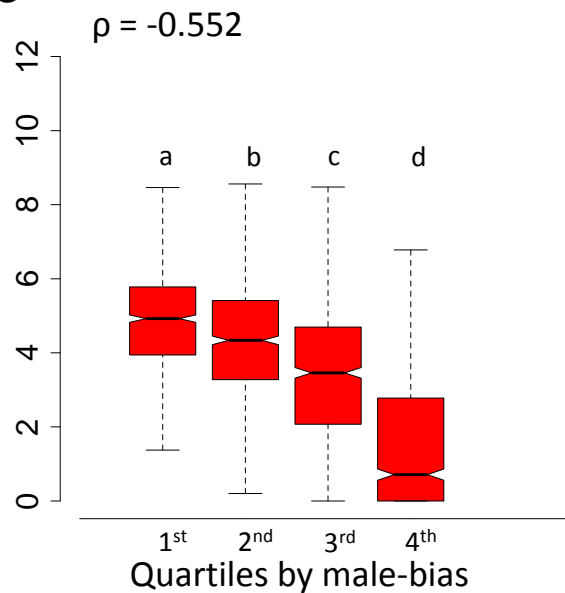**D**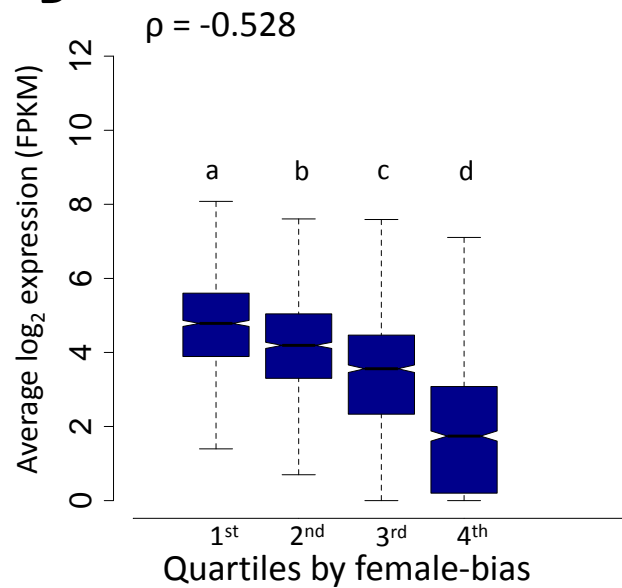**E**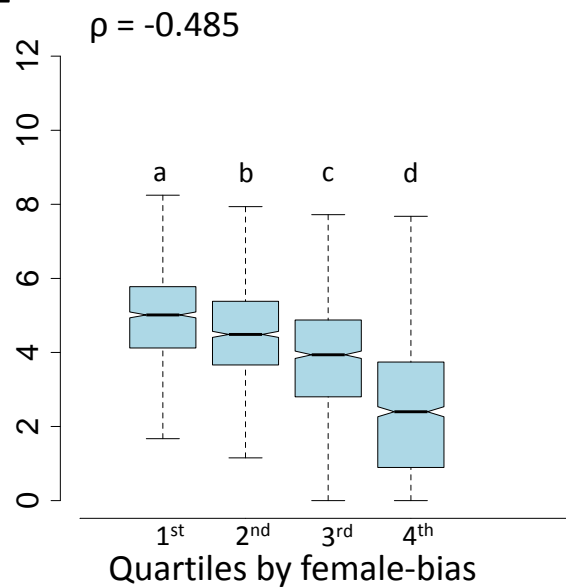**F**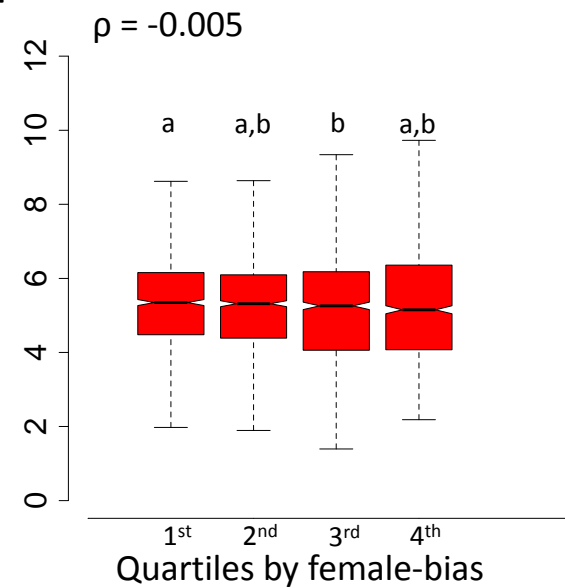

Supplement: Figure S5 — Expression level and sex-bias. Relationship between expression level and sex-bias for male-biased genes in dominant males (panel A), subordinate males (panel B) and females (panel C). Relationship between expression level and sex-bias for female-biased genes in dominant males (panel D), subordinate males (panel E) and females (panel F). Pairwise tests of significant difference between quartiles are denoted with letters, shared letters indicate that quartiles within a panel are not significantly different (Wilcoxon test, p<0.05). Spearman rank order correlations are given for each panel. (PDF) [file pgen.1003697.s005.pdf]
