# Supplementary material for: Masculinization of Gene Expression Is Associated with Exaggeration of Male Sexual Dimorphism
Source: PLoS Genet. 2013 Aug 15;9(8):e1003697. doi: 10.1371/journal.pgen.1003697 (PMC3744414; doi:10.1371/journal.pgen.1003697)
Supplement: Table S1 — Number of sex-biased autosomal genes expressed in the spleen, brain and gonad of the turkey. Genes are sex biased if they are expressed at least two-fold higher in one sex with an adj. p-value<0.05. (DOCX) [file pgen.1003697.s006.docx]

| Tissue | Number of expressed  autosomal genes | Number of  male-biased genes | Number of  female-biased genes |
| --- | --- | --- | --- |
| Spleen^A^ | 8560 | 0 | 0 |
| Brain^B^ | 9082 | 2 | 0 |
| Gonad^C^ | 9872 | 2217 | 2908 |

^A^Differential expression between the sexes was calculated by unpaired t-tests and adjusted for multiple testing using the Benjamini-Hochberg method based on expression data for two females and three dominant males.

^B^Differential expression was calculated with χ^2^ tests based on within-sex representative pools, each of two biological replicates, and *p*-values were then corrected for multiple testing using the Benjamini-Hochberg method.

^C^Differential expression was calculated by unpaired t-tests and adjusted for multiple testing using the Benjamini-Hochberg method based on expression data for five females and five dominant males.
