# Supplementary material for: Masculinization of Gene Expression Is Associated with Exaggeration of Male Sexual Dimorphism
Source: PLoS Genet. 2013 Aug 15;9(8):e1003697. doi: 10.1371/journal.pgen.1003697 (PMC3744414; doi:10.1371/journal.pgen.1003697)
Supplement: Table S2 — List of significantly different GO terms for genes shared between female and subordinate male turkeys. GO term enrichment analysis for 195 genes shared between female and subordinate males. (DOCX) [file pgen.1003697.s007.docx]

| GO terms Adjusted p-value |
| --- |
| \| transmembrane signaling receptor activity \| 3.61E-04 \| \| --- \| --- \| \| signaling receptor activity \| 4.52E-04 \| \| G-protein coupled receptor activity \| 3.63E-03 \| \| receptor activity \| 6.50E-03 \| \| signal transducer activity \| 2.35E-02 \| \| molecular transducer activity \| 1.96E-02 \| \| extracellular region \| 6.04E-06 \| \| proteinaceous extracellular matrix \| 1.59E-04 \| \| extracellular region part \| 1.13E-04 \| \| extracellular matrix \| 1.05E-04 \| \| plasma membrane \| 6.35E-03 \| \| intrinsic to membrane \| 0.0132 \| \| integral to membrane \| 0.013 \| \| membrane part \| 0.0114 \| |
