# Supplementary material for: Masculinization of Gene Expression Is Associated with Exaggeration of Male Sexual Dimorphism
Source: PLoS Genet. 2013 Aug 15;9(8):e1003697. doi: 10.1371/journal.pgen.1003697 (PMC3744414; doi:10.1371/journal.pgen.1003697)
Supplement: Table S3 — List of significantly different GO terms for genes shared between dominant and subdominant male turkeys. GO term enrichment analysis for 490 genes shared between dominant and subordinate males. (DOCX) [file pgen.1003697.s008.docx]

| GO terms Adjusted p-value |
| --- |
| \| substrate-specific channel activity \| 1.90E-09 \| \| --- \| --- \| \| passive transmembrane transporter activity \| 2.01E-09 \| \| channel activity \| 1.34E-09 \| \| ion channel activity \| 2.32E-09 \| \| cation channel activity \| 1.09E-07 \| \| ion gated channel activity \| 2.47E-07 \| \| gated channel activity \| 2.12E-07 \| \| transmembrane transporter activity \| 2.71E-06 \| \| ion transmembrane transporter activity \| 5.76E-06 \| \| substrate-specific transmembrane transporter activity \| 1.24E-05 \| \| transporter activity \| 1.76E-05 \| \| ligand-gated channel activity \| 4.59E-05 \| \| ligand-gated ion channel activity \| 4.23E-05 \| \| substrate-specific transporter activity \| 4.48E-05 \| \| extracellular ligand-gated ion channel activity \| 7.35E-05 \| \| cation transmembrane transporter activity \| 2.94E-04 \| \| metal ion transmembrane transporter activity \| 8.15E-04 \| \| voltage-gated cation channel activity \| 8.30E-04 \| \| inorganic cation transmembrane transporter activity \| 2.24E-03 \| \| transmembrane signaling receptor activity \| 3.01E-03 \| \| voltage-gated channel activity \| 3.24E-03 \| \| voltage-gated ion channel activity \| 3.09E-03 \| \| excitatory extracellular ligand-gated ion channel activity \| 6.19E-03 \| \| calcium channel activity \| 8.84E-03 \| \| C-C chemokine receptor activity \| 1.13E-02 \| \| C-C chemokine binding \| 1.09E-02 \| \| chemokine receptor activity \| 1.49E-02 \| \| G-protein coupled chemoattractant receptor activity \| 1.44E-02 \| \| potassium channel activity \| 1.80E-02 \| \| calcium ion transmembrane transporter activity \| 1.92E-02 \| \| glutamate receptor activity \| 2.66E-02 \| \| signaling receptor activity \| 2.63E-02 \| \| voltage-gated potassium channel activity \| 4.14E-02 \| \| potassium ion transmembrane transporter activity \| 4.70E-02 \| \| peptide receptor activity \| 4.77E-02 \| \| G-protein coupled peptide receptor activity \| 4.64E-02 \| \| cilium \| 8.64E-05 \| \| ion channel complex \| 2.96E-03 \| \| cell projection part \| 2.36E-03 \| \| synaptic membrane \| 4.52E-03 \| \| synapse \| 4.96E-03 \| \| postsynaptic membrane \| 4.89E-03 \| \| cation channel complex \| 1.00E-02 \| \| plasma membrane part \| 1.02E-02 \| \| axonemal dynein complex \| 1.19E-02 \| \| synapse part \| 1.28E-02 \| \| presynaptic membrane \| 1.48E-02 \| \| dynein complex \| 1.86E-02 \| \| cell projection \| 1.79E-02 \| \| plasma membrane \| 3.36E-02 \| \| intrinsic to membrane \| 3.91E-02 \| \| integral to membrane \| 3.78E-02 \| \| acrosomal vesicle \| 4.95E-02 \| \| terminal bouton \| 4.67E-02 \| \| ion transport \| 9.47E-06 \| \| ion transmembrane transport \| 3.04E-03 \| \| transmembrane transport \| 3.57E-03 \| \| regulation of ion transmembrane transport \| 1.90E-02 \| \| organic acid transport \| 1.56E-02 \| \| carboxylic acid transport \| 1.30E-02 \| \| regulation of transmembrane transport \| 1.62E-02 \| \| regulation of ion transport \| 1.55E-02 \| \| synaptic transmission \| 1.67E-02 \| \| single organism signaling \| 2.21E-02 \| \| signaling \| 2.01E-02 \| \| anion transport \| 2.43E-02 \| \| cation transport \| 2.68E-02 \| \| cell-cell signaling \| 3.31E-02 \| \| synaptic transmission, glutamatergic \| 4.44E-02 \| |
|  |

BENJAMINI, Y., and Y. HOCHBERG, 1995 Controlling the false discovery rate: a practical and powerful approach to multiple testing. Journal of the Royal Statistical Society Series B, 57: 289–300.
