# Supplementary material for: Masculinization of Gene Expression Is Associated with Exaggeration of Male Sexual Dimorphism
Source: PLoS Genet. 2013 Aug 15;9(8):e1003697. doi: 10.1371/journal.pgen.1003697 (PMC3744414; doi:10.1371/journal.pgen.1003697)
Supplement: Table S4 — List of genes differentially expressed between subordinate and dominant male turkeys. Binomial p-values, adjusted for multiple comparisons, were calculated in DESeq. An asterisk indicates the single differentially expressed gene found to lie within 10 kb of a testosterone receptor binding site. (DOCX) [file pgen.1003697.s009.docx]

|  | Gene | Fold change (log_2_ sm – dm) | Adjusted binomial *p*-value |
| --- | --- | --- | --- |
| Subordinate male-biased | *cytochrome P450, 11, A1 (CYP11A1)* | 1.61 | 0.0032 |
|  | *nidogen 1 (NID1)* | 1.50 | 0.0085 |
|  | *laminin, gamma 3 (LAMC3)* | 1.38 | 0.024 |
|  | *collagen, type V1, alpha 2 (COL6A2)* | 1.07 | 0.024 |
|  | *novel gene* | 1.29 | 0.037 |
|  | *DENN domain containing protein 5B (DENND5B)* | 1.02 | 0.049 |
| Dominant  male-biased | *short chain dehydrogenase/reductase family 9C, member 7 (SDR9C7)* | -3.22 | 0.0012 |
|  | *dual specificity phosphatase and pro isomerase domain containing 1 (DUPD1)* | -2.61 | 0.0012 |
|  | *transporter associated with antigen processing 2* | -1.42 | 0.0032 |
|  | *PHD finger protein 7 (PHF7)* | -1.56 | 0.0059 |
|  | *inositol 1,4,5-triphosphate receptor interacting protein-like 1 (ITPRIPL1)* | -1.52 | 0.0085 |
|  | *spermatogenesis associated 18 homolog (SPATA18)* | -1.47 | 0.013 |
|  | *phytanoyl-CoA 2-hydroxylase interacting protein-like (PHYHIPL)** | -1.44 | 0.024 |
|  | *leucine rich repeat containing 7 (LRRC7)* | -1.21 | 0.024 |
|  | *iron-sulfur cluster homolog (IBA57)* | -1.18 | 0.024 |
|  | *replication factor 4 (RFC4)* | -1.04 | 0.024 |
|  | *OTU deubiquinating enzyme 1 homolog (YOD1)* | -1.15 | 0.025 |
|  | *interleukin 18 receptor 1 (IL18R1)* | -1.51 | 0.025 |
|  | *C22orf23* | -1.24 | 0.025 |
|  | *t-complex-associated-testis-expressed 3*  *(TCTE3)* | -1.20 | 0.033 |
|  | *mitochondrial ribosomal protein L18* | -1.01 | 0.049 |
