# Supplementary material for: Masculinization of Gene Expression Is Associated with Exaggeration of Male Sexual Dimorphism
Source: PLoS Genet. 2013 Aug 15;9(8):e1003697. doi: 10.1371/journal.pgen.1003697 (PMC3744414; doi:10.1371/journal.pgen.1003697)
Supplement: Table S5 — Number and percentage of genes that reside in close proximity to at least one testosterone DNA binding motif and are male-biased. P-values are calculated using a two-sided Z-test by comparison to the actual number of male-biased genes in the genome (2217, 22.46%). (DOCX) [file pgen.1003697.s010.docx]

| **Receptor position** | **Male-biased** | ***p*-value** |
| --- | --- | --- |
| 2kb upstream | 48 (20.17%) | 0.403 |
| 5kb upstream | 116 (20.03%) | 0.174 |
| 10kb upstream | 234 (22.48%) | 0.988 |
