# Supplementary material for: Masculinization of Gene Expression Is Associated with Exaggeration of Male Sexual Dimorphism
Source: PLoS Genet. 2013 Aug 15;9(8):e1003697. doi: 10.1371/journal.pgen.1003697 (PMC3744414; doi:10.1371/journal.pgen.1003697)
Supplement: Table S6 — Average log2 fold change between subordinate and dominant males for genes that reside in close proximity to at least one predicted testosterone DNA binding motif. Significant difference from overall average log2 fold change (0.00044) was calculated by a permutation test with 1000 replicates. (DOCX) [file pgen.1003697.s011.docx]

| **Receptor position** | **Average log_2_ fold change (subordinate - dominant)** | ***p*-value** |
| --- | --- | --- |
| 2kb upstream | 0.0178 | 0.293 |
| 5kb upstream | 0.0149 | 0.256 |
| 10kb upstream | 0.00194 | 0.462 |
